# Isoniazid subverting erythrocyte homeostasis: implications for tuberculosis therapy

**DOI:** 10.3389/fphar.2026.1721617

**Published:** 2026-02-09

**Authors:** Muhammad Sikandar, Maria Fatima, Kashif Jilani, Li Xing

**Affiliations:** 1 Institutes of Biomedical Sciences, Shanxi University, Shanxi, China; 2 Department of Biochemistry, University of Agriculture, Faisalabad, Pakistan

**Keywords:** amlodipine, erythrocyte toxicity, hemolysis, isoniazid, *Mycobacterium tuberculosis* (MTB), oxidative stress

## Abstract

Isoniazid (INH) is a frontline anti-tuberculosis drug. Understanding the molecular mechanisms by which INH affects antioxidant defence in human blood cells, particularly erythrocytes vulnerable to oxidative damage, remains essential to improving therapy safety. Here, the transcriptomic data of tuberculosis INH therapy-treated HepG2 cell line were analyzed to identify differentially expressed genes (DEGs). DEGs were cross-referenced with curated oxidative stress (OS) gene sets from GeneCards, and protein-protein interaction (PPI) networks were constructed to identify hub OS genes associated with INH treatment-induced OS. Biochemical assays assessed antioxidant enzyme activities (SOD, GPx, CAT and ROS), erythrocyte morphology, membrane integrity, and calcium involvement following INH exposure *in vitro*. A total of 7202 DEGs were identified, with 196 overlapping OS-related genes forming a focused gene set. Key hub genes, including SOD1, SOD2, and GPx family members, were downregulated, corresponding to decreased antioxidant enzyme activities in erythrocytes exposed to INH (3–6 mM). Functional analysis highlighted upregulation of oxidative stress response pathways and upregulation of cell adhesion/survival pathways such as the IL17 signalling pathway. INH induced erythrocyte membrane blebbing and mean cell volume expansion, which was attenuated by calcium channel blockade, indicating Ca^2^-dependent mechanisms driving membrane destabilization. Haemolysis assays confirmed concentration-dependent erythrocyte fragility. The results show that INH may disrupt erythrocyte redox balance by suppressing critical antioxidant enzymes and activating OS pathways, leading to cellular dysfunction and membrane instability mediated by calcium influx. These findings integrate transcriptomic insights with biochemical validation, underscoring the importance of monitoring oxidative damage in patients undergoing INH therapy.

## 1 Introduction

The mature red blood cells (RBC) vastly outnumber all other cell types in mammals (Lam et al., 2021). Consequently, RBC damage severely compromises health, leading to complex syndromes (Sundd et al., 2019; Panch et al., 2019). Persistent research on hemolysis follows the classical paradigm of intravascular hemolysis via complement activation by blood group antibodies targeting RBC antigens (Jäger et al., 2019). This activation generates cleavage products and assembles the membrane attack complex (MAC) (Jäger et al., 2019). The MAC forms transmembrane pores in the RBC membrane, disrupting its integrity and causing osmotic lysis (Risitano et al., 2014).

Tuberculosis (TB) caused by *Mycobacterium tuberculosis* (*Mtb*) remains the deadliest infectious disease worldwide, with an estimated loss of 1.32 million lives in 2024 (Orga and nization, 2025). *Mtb* infection triggers intense oxidative stress (OS) in RBCs, overwhelming antioxidant defenses and damaging hemoglobin and spectrin. This compromises membrane integrity, causing hemolysis, anemia, and the release of harmful molecules including heme/iron (Mapamba et al., 2025). INH is a frontline antibiotic primarily used to treat TB (Lei et al., 2000). INH is a prodrug that requires activation by the bacterial catalase peroxidase enzyme *KatG* (Lei et al., 2000). However, some clinical studies confirm that INH can induce OS caused by hemolysis, particularly in TB patients with G6PD deficiency (Hernandez-Ochoa et al., 2022; Youngster et al., 2010). INH metabolites deplete glutathione and generate ROS, overwhelming erythrocyte antioxidant defenses (Zentner et al., 2020). Furthermore, INH-induced toxicity is known to be exacerbated in patients with comorbidities (HIV, diabetes) and enzyme polymorphisms (NAT2, GSTA2), which increases their susceptibility to OS and impairs detoxification (Zentner et al., 2020).

OS exhibits a dual role during INH treatment and *Mtb* infection. While reactive oxygen species (ROS), like superoxide radicals, are essential for activating INH through *KatG*-mediated conversion into its effective form (Bulatovic et al., 2002). Nrf2/ARE impairment drives ROS buildup and cell injury, promoting *Mtb* survival while upregulating antioxidants against INH/immune OS, potentially causing liver dysregulation (de Vries et al., 2008). Furthermore, the liver is severely affected by INH, primarily through the generation of damaging ROS in its cells (Deng et al., 2025). This hepatic OS propagates systemically to erythrocytes, inducing eryptosis, a programmed suicidal death pathway as the primary mechanism precipitating anemia in chronic liver disease and hepatic failure (Tkachenko et al., 2025). The impaired liver function leads to the accumulation of circulating toxins, such as bilirubin, which are potent inducers of eryptosis (Mei et al., 2022).

By using multiple computational methods, we examined the transcriptomic results of INH-treated HepG2 cell line and identified key DEGs associated with INH-induced OS and then experimental assays validated our computational analysis.

## 2 Materials and methods

### 2.1 Datasets

The gene expression profiles of HepG2 cells were analyzed, comparing three untreated samples to three samples treated with INH (GSE168473, NCBI Gene Expression Omnibus, GEO).

### 2.2 Screening differentially expressed genes (DEGs)

The GEO2R tool (https://www.ncbi.nlm.nih.gov/geo/geo2r/) was used to identify DEGs (Barrett et al., 2012). DEGs were identified using empirical Bayes moderation (|log2FC| > 1, adj. p < 0.05), with Benjamini–Hochberg FDR control for statistical reliability (Colaprico et al., 2016).

### 2.3 Identification of key OS-related DEGs through integration of GeneCards curation and transcriptomic data

To enhance DEG reliability, we compiled a curated list of experimentally validated OS-associated human genes from GeneCards (https://www.genecards.org/). The filtered human gene list was intersected with HepG2 DEGs to produce a final curated set of OS-associated genes using a Venn diagram approach (http://bioinformatics.psb.ugent.be/webtools/Venn/). Genes overlapping between HepG2 transcriptomic data and the filtered GeneCards list were designated key OS-related DEGs (OS-DEGs).

### 2.4 PPI network and cytoscape analysis

PPI network for key OS-DEGs or top hub OS-DEGS were constructed using the STRING database (v12.0; https://www.string-db.org) (Szklarczyk et al., 2023). Interactions were filtered with high-confidence criteria: interaction score ≥0.700, ≥2 interactions per node, and FDR ≤0.05. Cytoscape (v3.10.3) was used to identify critical modules and hub genes (Shannon et al., 2003). The top 20 hub genes were prioritized using CytoHubba’s maximal clique centrality (MCC) algorithm.

### 2.5 Functional enrichment analysis

To determine the functions of the identified OS-DEGs, Gene Ontology (GO) and Kyoto Encyclopedia of Genes and Genomes (KEGG) enrichment analysis including Wiki and Reactome pathway analysis were performed on OS-DEGs. GO analysis categorizes terms into biological processes (BP), molecular functions (MF), and cellular components (CC) pathways. Functional annotation and enrichment analysis were conducted using the Database for Annotation, Visualization and Integrated Discovery (DAVID) alongside the Enrichr tool, accessible at https://maayanlab.cloud/Enrichr/. A significance threshold of an adjusted p-value ≤0.05 was applied. The top ten most significantly enriched terms from the GO database and the KEGG pathways, selected based on the highest gene counts for further investigation (Huang et al., 2007; Mering et al., 2003).

### 2.6 Gene set enrichment analysis (GSEA)

A GSEA pathway network was constructed to visualize the relationships between the enriched pathways and the hub genes identified in the analysis. The network was generated using the networkx Python library and visualized with matplotlib (Larsen et al., 2017; Yim et al., 2018). Pathways were considered significantly enriched based on a false discovery rate (FDR) < 0.25 and a normalized enrichment score (NES) threshold.

### 2.7 OS hub gene-disease associations

We performed a DisGeNET meta-analysis to predict OS-specific hub gene-disease associations (Piñero et al., 2015) by using Network Analyst (Zhou et al., 2019). Furthermore, the target genes of interest are mapped to the disease-specific INH-OS. Disease degree was calculated for each hub target based on nodes and edges, where nodes denote hub targets and diseases, and edges represent their interaction strength the greater the interaction, the higher the edge value.

### 2.8 Biochemical study design

Our study isolated the direct effects of INH on erythrocytes to evaluate its role in OS independent of confounding variables from *Mtb* infection. Erythrocytes were selected due to their heightened vulnerability to OS, which can compromise their antioxidant defense mechanisms, particularly enzymes such as superoxide dismutase (SOD), glutathione peroxidase (GPx), and catalase (CAT) critical for maintaining intracellular redox equilibrium along with quantification of ROS level.

### 2.9 Concentration of INH determination

The INH concentrations were selected to model the heightened systemic oxidative challenge experienced by RBCs in patients with impaired detoxification, an approach validated in comparative toxicological studies (Zou et al., 2017). Furthermore, high-dose INH is commonly used in rodent models to simulate the metabolic stress relevant to human toxicity (Metushi et al., 2012).

### 2.10 Determination of SOD activity

SOD activity was measured in erythrocyte lysates. Blood samples (0.5 mL) were centrifuged (1100 × g, 10 min). The erythrocyte pellet was washed four times with saline, lysed in cold redistilled water (2 mL, 4 °C, 10 min), and diluted 100-fold in PBS (pH 7.0). Activity was quantified using the Ransod kit (Randox Laboratories, United Kingdom), measuring inhibition of superoxide radical-mediated reduction of nitroblue tetrazolium (NBT) to formazan at 560 nm (DU65 spectrophotometer, Beckman, Germany). Assays were performed in triplicate and normalized to the total protein.

### 2.11 Determination of GPx activity

GPx activity was measured using the Ransel kit (Randox Laboratories, Crumlin, County Antrim, United Kingdom) based on a UV method proposed by Paglia and Valentine (Paglia and Valentine, 1967). GPx activity in heparinized whole blood was measured by monitoring NADPH consumption at 470 nm (Beckman spectrophotometer) during the enzymatic oxidation of glutathione.

### 2.12 Determination of CAT activity

CAT activity was assayed following established protocols (Sicińska et al., 2020; Aebi, 1984). Briefly, 100 µL of sample and 100 µL of reaction mixture composed of 50 mM phosphate buffer (pH 7.0) and 54 µL of 5.9 nM hydrogen peroxide (H_2_O_2_) diluted in 10 mL of water were added to a 96-well plate. CAT activity was quantified by measuring the rate of H_2_O_2_ decomposition via absorbance at 240 nm using an ELISA plate reader.

### 2.13 Measurement of mean cell volume (MCV)

MCV and other erythrocyte indices were determined using a Technicon H*3 hematology analyzer (Tarrytown, NY, United States) following established protocols (d'Onofrio et al., 1995). The analyzer automatically quantified RBC count, reticulocyte count, MCV (for both mature RBCs and reticulocytes), and hemoglobin (Hb) content/concentration.

### 2.14 Ca^+2^ assay

To investigate Ca^2+^-mediated mechanisms in anti-TB drug-induced eryptosis, erythrocytes were isolated from fresh blood via centrifugation. After removing the plasma (upper layer) and buffy coat (leukocytes/platelets, middle layer), the erythrocyte pellet was washed repeatedly with physiological saline to remove residual plasma. Purified erythrocytes were co-treated with 10 µM amlodipine (Ca^2+^ channel blocker; Teva Pharmaceutical Industries, Israel) and INH to assess Ca^2^-dependent apoptotic pathways (Rana et al., 2019).

### 2.15 Hemolysis measurement

Erythrocytes were incubated with test compounds (37 °C, specified duration). After gentle centrifugation (20 × g, 3 min, 25 °C), supernatant hemoglobin release (indicating hemolysis) was measured at 405 nm. INH-induced hemolysis (%) was calculated *versus* a 100% lysis control (deionized water-lysed cells) (Lupescu et al., 2014).

### 2.16 Determination of ROS production

ROS levels were quantified using the fluorescent probe 2′,7′-dichlorodihydrofluorescein diacetate (DCFDA). Cells in FACS buffer were analyzed by flow cytometry on a FACSCalibur system (Becton Dickinson, Heidelberg, Germany). The DCFDA-derived fluorescence was detected in the FL-1 channel, with excitation set at 488 nm and emission collected at 530 nm. The cells were analyzed based on FL1-Height fluorescence intensity measured on a logarithmic scale (Sinha et al., 2006).

### 2.17 INH-induced erythrocyte OS in TB therapy-an epidemiological evidence synthesis

A systematic search using keywords like “isoniazid,” “erythrocyte,” “oxidative stress,” and “hematological profile” identified clinical studies on TB patients receiving standard INH-containing therapy that quantitatively assessed erythrocyte parameters, such as antioxidant enzymes (SOD, CAT, GPx) or hematological indices (MCV, Hgb, RDW), before and after treatment (Vidhya et al., 2019; Meca et al., 2021; Kassa et al., 2016). The extracted data were then compiled into a comparative table to demonstrate the *in vivo* impact of INH on the circulating erythrocyte population.

### 2.18 Statistical analysis

Experiments were conducted in three independent biological replicates under standardized conditions. Data are expressed as mean ± SEM. Statistical significance was analyzed by one-way ANOVA with Tukey’s post-hoc test.

## 3 Results

### 3.1 Identification of OS-DEGs in response to INH

**FIGURE 1:**
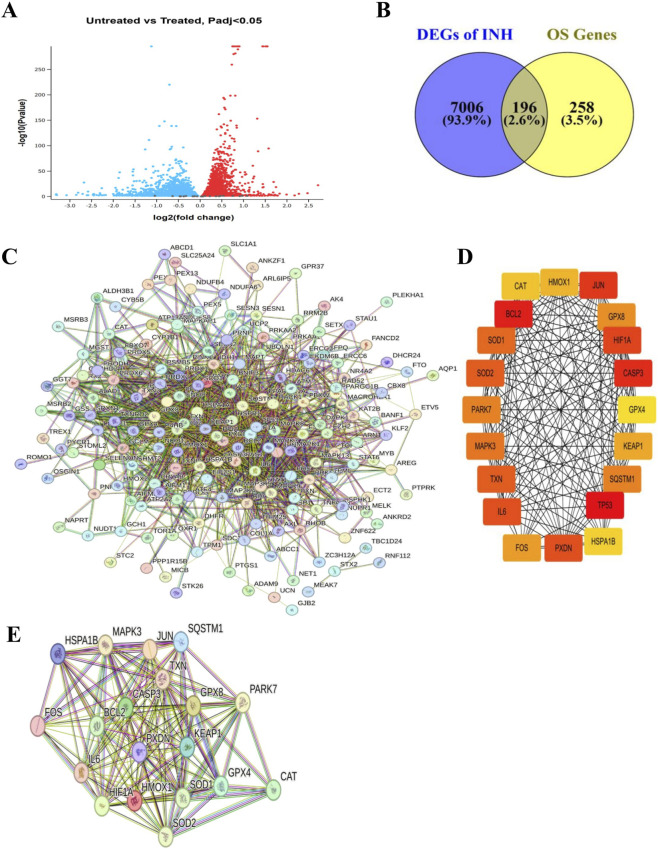
Identification of oxidative stress-related differentially expressed genes in response to INH. **(A)** Volcano plot of differential gene expression in HepG2 cells (GSE168473; three untreated vs. Three INH-treated samples), generated using GEO2R with empirical Bayes moderation (|log2FC| > 1, adj. p < 0.05, Benjamini–Hochberg FDR control). The x-axis shows log2 fold change (FC); the y-axis shows -log10 adjusted p-value. Red dots indicate significantly upregulated genes (adj. p < 0.05, FC ≥ 2); blue dots indicate significantly downregulated genes (adj. p < 0.05, FC ≤ 0.5); gray dots are non-significant (adj. p ≥ 0.05 or 0.5 < FC < 2). **(B)** Venn diagram identifying overlapping genes that are differentially expressed (p < 0.05) in INH-treated vs. untreated HepG2 cells and curated as experimentally validated OS-associated from GeneCards, yielding key OS-DEGs. **(C)** Protein-protein interaction (PPI) network of 196 key OS-DEGs, constructed in the STRING database (v12.0; high-confidence interactions: score ≥0.700, ≥2 interactions/node, FDR ≤0.05). **(D)** Hub genes within the PPI network, prioritized by CytoHubba’s maximal clique centrality (MCC) algorithm (top 20 shown), with GPX8, SOD2, TXN, SOD1, and HMOX1 exhibiting the highest connectivity and visualized in Cytoscape (v3.10.3). **(E)** PPI network was generated to map the functional associations among the top 20 hub genes identified from OS-DEGs in STRING database (v12.0; high-confidence interactions: score ≥0.700, ≥2 interactions per node, FDR ≤0.05).

The analysis of transcriptomic results of INH-treated HepG2 cells compared to untreated controls revealed 7,202 DEGs out of a total of 16,921 genes (Figure 1A). Cross-referencing the DEGs with a curated OS gene set from GeneCards identified 196 overlapping OS-DEGs (Figure 1B). This gene subset identifies key molecular players in the INH-induced OS response, providing a basis to investigate the dysregulated redox signaling and potential therapeutic targets.

### 3.2 Protein-protein interactions (PPI) highlight key OS hub genes associated with antioxidant enzyme regulation

Building a PPI network from 196 OS-DEGs using STRING (Figure 1C) and analyzing the centrality of these genes via Cytoscape revealed 20 critical hub genes central to OS defense and antioxidant enzyme regulation (Figure 1D). These include antioxidant enzymes such as CAT, SOD1, SOD2, GPx4, GPx8, and transcriptional regulators (TP53, FOS, JUN). A secondary PPI network of 20 hub genes shown in Figure 1D isolated the core OS response module, excluding peripheral interactions to highlight key antioxidant and regulatory elements against INH-OS stress (Figure 1E). These hub genes represent vital nodes in redox homeostasis and cellular responses to INH-induced oxidative challenges.

### 3.3 Functional enrichment reveals OS-related pathways underlying disrupted redox homeostasis

To understand the biological roles of the significant hub genes, we performed a comprehensive pathway enrichment analysis. This analysis maps the gene set to key biological processes, cellular compartments, and molecular functions using GO (BP, CC, MF), KEGG pathways, WikiPathways, and Reactome, providing a comprehensive view of their functional impact.

**FIGURE 2:**
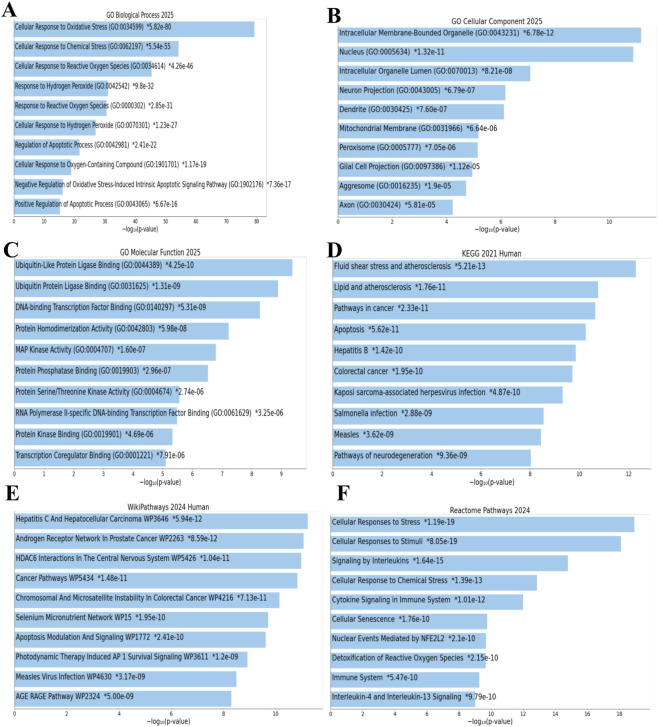
Functional enrichment reveals OS-related pathways underlying disrupted redox homeostasis. Functional enrichment analyses of OS-DEGs (prioritized by CytoHubba’s MCC algorithm), performed using DAVID and Enrichr (maayanlab.cloud/Enrichr/) with adjusted p-value ≤0.05. Top 10 terms/pathways per category selected by highest gene counts. **(A)** GO biological process (BP) analysis. **(B)** GO cellular component (CC) analysis. **(C)** GO molecular function (MF) analysis. **(D)** KEGG pathway analysis identified “Fluid shear stress and atherosclerosis” as the most enriched. **(E)** WikiPathways showed “Hepatocellular carcinoma” as the top pathway. **(F)** Reactome pathways confirmed enrichment in “Cellular responses to stress” and detoxification pathways.

Within the BP category, significant enrichment was observed in pathways related to the cellular response to OS, followed by cellular responses to chemical stress and ROS (Figure 2A). For the CC category, the CC pathway analysis showed enrichment in intracellular membrane organelles, nucleus, and organelle lumens (Figure 2B). In the MF category, DNA-binding transcription factors were predominantly enriched, including RNA polymerase II-specific DNA-binding transcription factor activity, ubiquitin-protein ligase binding, and sequence-specific DNA binding at core promoter regions (Figure 2C).

KEGG pathway analysis identified the fluid shear stress and atherosclerosis pathway as the most significantly enriched, followed by pathways related to lipid metabolism and atherosclerosis, cancer, and apoptosis (Figure 2D). In the WikiPathways analysis, the hepatocellular carcinoma pathway was the most enriched, with additional enrichment observed in the androgen receptor network in prostate cancer and HDAC6 interactions in the central nervous system (Figure 2E). Reactome pathway analysis highlighted the cellular response to stress as the primary enriched pathway, along with cellular responses to stimuli, interleukin signaling, and cellular response to chemical stress (Figure 2F).

### 3.4 GSEA reveals suppression of survival and adhesion pathways, complementing OS stress

**FIGURE 3:**
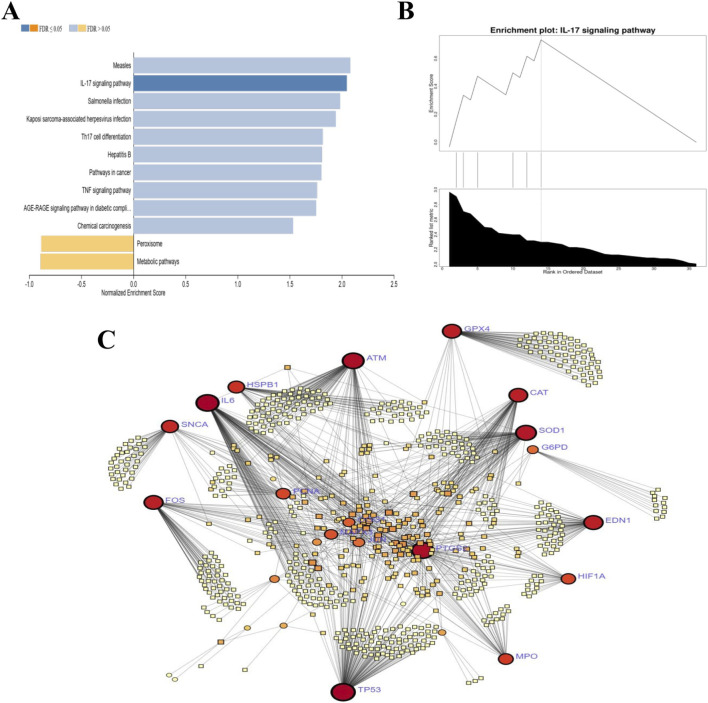
Integrated gene and pathway network analyses of the hub OS-DEGs. **(A)** GSEA pathway network of HepG2 OS-DEGs against KEGG database, constructed using networkx Python library and visualized with matplotlib; pathways shown with normalized enrichment score (NES) and FDR <0.25 (upregulated: positive NES, blue bars; downregulated: negative NES, orange bars). **(B)** GSEA enrichment plot of the IL-17 signaling pathway in INH-treated HepG2 cells, demonstrating significant upregulation and immune response activation post-INH exposure. **(C)** Disease-gene association network from DisGeNET meta-analysis via Network Analyst, mapping hub OS-DEGs (nodes) to OS-related diseases (nodes) with edge weights reflecting interaction strength/disease degree, highlighting gene pleiotropy and shared mechanisms relevant to INH-OS therapy.

GSEA analysis revealed significant alterations in several key pathways, including both upregulated and downregulated biological processes (Figure 3A). Notably, the IL-17 signaling pathway was identified as significantly upregulated, suggesting an activation of immune responses (Figure 3B). This is further supported by the upregulation of several infection-related pathways, including Measles, *Salmonella* infection, Kaposi sarcoma-associated herpesvirus infection, Hepatitis B, Th17 cell differentiation, TNF signaling pathway, AGE-RAGE signaling pathway in diabetic complications, and chemical carcinogenesis. Conversely, peroxisome and metabolic pathways were significantly downregulated, signalling shifts in energy metabolism. These changes reflect coordinated immune-inflammatory responses alongside OS effects, indicating systemic alterations during INH exposure.

### 3.5 Disease-gene network correlates hub OS-DEGs with multi-disease associations underlying cellular stress mechanisms

The disease-gene association network highlights hub genes including GPx4, SOD1, SOD2, ATM, CAT, TP53, SNCA, FOS, G6PD, IL6, EDN1, HSPB1, and MPO as critical hubs involved in OS-related diseases (Figure 3C). GPx4 prevents lipid peroxidation and ferroptosis, linking OS to neurodegeneration, ischemia-reperfusion injury, and inflammation. SOD1 contributes to ALS, ischemic heart disease, and mitochondrial damage. SOD2 associates with mitochondrial dysfunction in Alzheimer’s and Parkinson’s. ATM mediates DNA damage response under OS, tied to neurodegeneration and cancer. CAT detoxifies H2O2, impacting cardiovascular disease, diabetes, and aging disorders.; TP53 drives OS responses in cancers and aging; SNCA causes oxidative neuronal injury in Parkinson’s; FOS contributes to OS pathways in cancer and inflammation; G6PD deficiency causes hemolytic anemia due to oxidative vulnerability; IL6 is a mediator of inflammation and OS in autoimmune and chronic inflammatory diseases; EDN1 contributes to oxidative damage in cardiovascular and kidney diseases; HSPB1 maintains redox homeostasis in neurodegeneration and cancer; MPO generates oxidants driving atherosclerosis, chronic inflammation, and neurodegeneration. These genes underscore pleiotropic roles in oxidative damage and cellular stress-related disease phenotypes.

Based on the conclusion of transcriptomic data, derived from the INH-treated HepG2 human liver cell line, we identified OS-related genes and pathways affected by INH treatment, which are essential for maintaining cellular redox balance. Hepatocytes serve as the primary site of INH bioactivation, where the drug is metabolized by catalase-peroxidase and cytochrome P450 2E1 into reactive metabolites (hydrazine, acetylhydrazine) and ROS (Tafazoli et al., 2008; Boelsterli and Lee, 2014). The transcriptomic analysis captured upstream OS pathway dysregulation occurring during this hepatic metabolism. While erythrocytes lack transcriptional machinery, they are highly vulnerable to circulating reactive metabolites and oxidative species generated from hepatic INH processing (Kumar et al., 2022; Bhadauria et al., 2007). The antioxidant genes identified in HepG2 cells encode proteins (SOD, GPx, CAT) that are evolutionarily conserved and functionally critical in erythrocytes (Bołdys et al., 2024). Therefore, we hypothesized that OS pathways disrupted transcriptionally in hepatocytes would manifest as functional impairment of antioxidant enzyme activities in erythrocytes. Building on these transcriptomic insights, we proceeded to experimentally investigate the effects of INH on human erythrocytes, a cell type highly susceptible to OS due to their oxygen-transport function and reliance on antioxidant enzymes for protection.

### 3.6 INH-OS: dose-dependent SOD/GPx/CAT suppression impairs RBC ROS/lipid peroxide clearance

**FIGURE 4:**
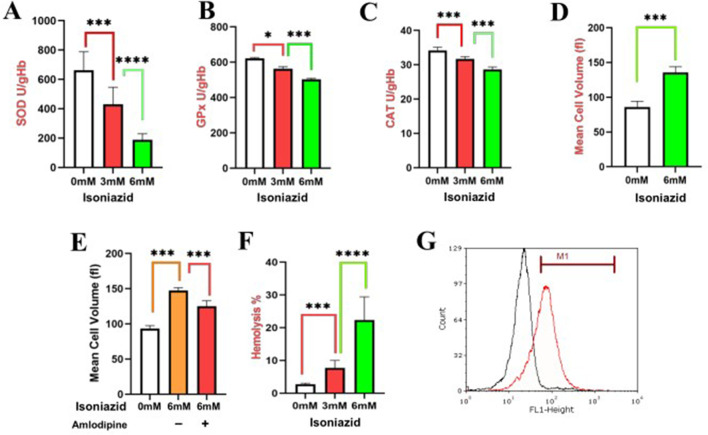
Effects of INH on antioxidant enzymes in erythrocytes. INH-induced oxidative stress and eryptosis in isolated human erythrocytes (3 mM/6 mM INH, 48 h incubation at 37 °C; n = 6 independent replicates per experiment; one-way ANOVA with Tukey’s post-hoc test; data as mean ± SEM; *, *p* < 0.05; ***, *p* < 0.001; ****, *p* < 0.0001). **(A)** SOD activity suppression (Ransod kit; NBT reduction inhibition at 560 nm). **(B)** GPx activity reduction (Ransel kit; NADPH consumption at 470 nm). **(C)** Dose-dependent CAT impairment (H_2_O_2_ decomposition at 240 nm). **(D)** MCV expansion and membrane blebbing (Technicon H3 analyzer). **(E)** Amlodipine (10 μM Ca^2+^ channel blocker) inhibition of INH-induced MCV expansion. **(F)** Dose-responsive hemolysis (% Hb release at 405 nm vs. 100% lysis control). **(G)** ROS levels were quantified using the DCFDA probe; cells in FACS buffer were analyzed on a FACSCalibur (FL-1 channel: 488 nm excitation, 530 nm emission; log-scale FL1-Height). Histograms show control (black) peaking at ∼10^1^ units (basal ROS) vs. INH-treated (red, 6 mM, 48 h) shifting to ∼10^(61)-10^2^ units with clear separation, confirming OS induced eryptosis.

INH treatment caused significant dysregulation of genes associated with OS in the human hepatoma HepG2 cell line (Figures 1–3). This dysregulation involve increased ROS production, altered antioxidant enzyme levels, and disruption of mitochondrial function. We then tested the effects of INH on the OS of erythrocytes. INH exposure (3 mM and 6 mM, 48 h) significantly impaired erythrocyte antioxidant defenses in a concentration-dependent manner by markedly reducing activity of SOD (Figure 4A), GPx (Figure 4B), and CAT (Figure 4C), providing direct experimental validation of the transcriptional dysregulation of their encoding genes predicted by HepG2 omics analysis, confirming the biological relevance of the identified OS pathways.

### 3.7 INH exposure triggers erythrocyte membrane blebbing and MCV expansion

As shown in Figure 4D, treatment of erythrocytes with INH for 48 h resulted in an elevation in MCV, most notably at 6 mM. This morphological alteration correlated with membrane blebbing, a phenomenon driven by aberrant intracellular calcium (Ca^2+^) accumulation, which compromises cytoskeletal stability and promotes cellular swelling. To probe Ca^2+^’s involvement, pre-treatment with calcium channel inhibitor amlodipine attenuated INH-induced MCV expansion (Figure 4E), implicating Ca^2+^ influx in membrane destabilization. Hemolysis assays (Figure 4F) showed dose-dependent Hb release, heightened at 6 mM INH, confirming membrane disruption. INH (6 mM, 48 h) induced ROS overproduction as histograms showed control (black) peaking at ∼10^1^ FL1-Height (basal ROS) vs. 6 mM INH-treated (red) shifting to ∼10^1^·^5^–10^2^ units, with clear separation. This ROS surge evidences OS, supporting eryptotic signaling via cell blebbing (Figure 4G).

### 3.8 Clinical haematological disruptions during INH-containing anti-tuberculosis therapy (ATT)

In cohort studies of TB patients on INH-containing ATT, oxidative damage and haemolysis cause erythrocyte changes: hemoglobin/hematocrit drops, RDW rise (13.5%–31.1%), MCV increase (20.9% by 6 months), and persistent anemia (88.9%). Erythrocyte haemolysis was observed under INH therapy (detailed in Supplementary Tables S1 and S2).

## 4 Discussion

Understanding the molecular and cellular mechanisms underlying the OS is essential for improving treatment safety and efficacy. This study identified and then validated OS-DEGs in HepG2 cells and INH treatment in erythrocyte, specifically for anti-oxidant genes, linking transcriptomic alterations to functional impairments. These findings provide critical information for the mechanisms underlying the clinical observations during INH therapy (Supplementary Tables S1 and S2). The transcriptomic analysis identified 196 OS-DEGs in INH-treated HepG2 cells with hub genes such as SOD1, SOD2, GPx4, and CAT (Figures 1B,C), showing INH’s capacity to enhance ROS formation and inhibit antioxidant defenses. Moreover, HepG2 transcriptomics reveal upstream OS pathway dysregulation in the liver (primary site of INH metabolism). Erythrocyte enzyme impairment validates the downstream functional consequence. The critical link is the circulating reactive metabolites (e.g., hydrazine, ROS) generated by the HepG2 cells/liver, which subsequently attack the highly susceptible, non-nucleated erythrocytes (Preziosi, 2007; Luzzatto et al., 2016). Previous studies show that INH induces OS by generating free radical metabolites via cytochrome P450 enzymes like CYP2E1, which lead to excess ROS in hepatocytes, triggering oxidative damage (Sankar et al., 2024; Choi et al., 2002). The PPI network of 20 hub genes (Figure 1E), including TP53, FOS, JUN, and antioxidant enzymes, reveals INH-disrupted OS signaling pathways, surpassing prior isolated enzyme studies (Nhung et al., 2025). This prior study aligns with our findings of disrupted antioxidant enzyme expression and activity, showing significant SOD, GPx, and CAT reductions in INH-exposed erythrocytes (Figures 4A–C).

Furthermore, our functional enrichment analysis (Figures 2, 3A,B) identified significant activation of pathways related to OS responses, apoptosis regulation, and inflammatory signaling such as IL-17. These results highlight that INH may suppress the critical antioxidant response element (ARE)-mediated detoxification pathways (Meng et al., 2022). In addition, INH suppresses ERK1 phosphorylation in hepatocytes, preventing Nrf2 from activating and moving to the nucleus (Zhang et al., 2020), which blocks the protective antioxidant response, leading to increased OS, inflammation, and liver injury driven by IL-17 and other cytokines (Verma et al., 2015). Although, in RBCs, chronic INH exposure impairs Nrf2 antioxidant defenses, causing oxidative and membrane damage plus hemolysis. IL-17-driven inflammation exacerbates this, promoting anemia, shortened RBC lifespan, and liver injury (Hassan et al., 2016), which also corroborates our transcriptomic insight into impaired antioxidant gene regulation. Moreover, the systemic immune-inflammatory pathways enriched in our data correspond to reports that INH activates inflammatory cascades alongside redox imbalance, contributing to hepatocyte apoptosis and tissue injury (Zhuang et al., 2022; Gong et al., 2022).

Further mechanistic insights from a prior study complement our data (Figure 2). For instance, INH induces mitochondrial dysfunction by inhibiting respiratory chain enzymes, opening mPTP, and disrupting biogenesis regulators like PGC1α, resulting in energy depletion and apoptosis (Zhang et al., 2017). ROS overproduction, mitochondrial damage, and apoptosis align with our GSEA (Figures 3A,B) and PPI network (Figure 1C), indicating redox pathway dysregulation.

Disease-gene network analysis shows hub genes STAT1, HIF1A, and SOD1 link to multiple diseases, underscoring their pleiotropic roles in cellular stress responses across TB, heart failure, and cancer (Zhang et al., 2025). Moreover, HepG2 transcriptomic analysis revealed INH-disrupted OS signaling via JUN/FOS (AP-1) and antioxidant enzymes (CAT, SOD1/2, GPx), aligning with disease-gene network findings linking OS hubs to hematological pathology through G6PD (Figure 3C), a known hemolytic anemia risk factor exacerbated by INH in deficient patients (Chen et al., 2013). This molecular finding directly correlates with the established clinical observation that patients with G6PD deficiency are highly susceptible to INH-induced hemolysis (Chen et al., 2013). Thus, erythrocytes lacking transcriptional machinery for new antioxidant enzymes are highly vulnerable to ROS from hepatic INH metabolism (Supplementary Table S1). Experimentally, dose-dependent suppression of SOD, GPx, and CAT in human erythrocytes (Figures 4A–C) validates molecular findings, showing INH directly impairs antioxidant defenses. This matches reports of INH elevating oxidative damage and lipid peroxidation, disrupting redox homeostasis and RBC vulnerability (Zentner et al., 2020). Antioxidant dysfunction correlates with erythrocyte fragility via MCV increases and blebbing (Figure 4D), linked mechanistically to aberrant Ca2^+^ influx. Amlodipine protects against MCV expansion (Figure 4E), aligning with OS-induced Ca2^+^-mediated cytoskeletal destabilization, blebbing, and hemolysis (Figure 4F) (Salehi et al., 2012). INH induces significant OS in erythrocytes, evidenced by elevated intracellular ROS and eryptotic blebbing (Figure 4G). These findings align with prior reports of INH anti-*Mtb* drug promoting RBCs ROS generation and hemolytic damage (Kassa et al., 2016). Prior studies link ROS-induced eryptosis to Ca^2+^ influx and phosphatidylserine exposure. Our data confirm OS as the central mediator of INH cytotoxicity, with excess ROS driving membrane blebbing and apoptotic progression (Peter et al., 2016; Lang et al., 2006).

Collectively, our data show that INH not only disrupts the transcriptional control of antioxidant enzymes (JUN/FOS) (Figure 3C), but also functionally impairs the activity of the G6PD-dependent GPx system in erythrocytes. This dual hit provides a clear molecular mechanism for the observed clinical hemolysis. In addition, the INH concentrations (3–6 mM) used in our study define the mechanistic threshold for INH-induced OS, modeling elevated systemic ROS exposure in vulnerable patients such as those with G6PD deficiency or slow acetylator status who exhibit impaired drug metabolism, which is a standard approach for toxicological studies (Metushi et al., 2012).

The integrated HepG2 transcriptomics and experimental erythrocyte validation robustly links molecular changes to INH-induced cellular dysfunction. INH treatment transcriptionally dysregulates key OS pathways and hub genes (SOD1, GPx4, CAT) in the HepG2 liver cell line, reflecting the upstream metabolic stress. Crucially, we validated transcriptional dysregulation in circulating erythrocytes as functional impairment: dose-dependent antioxidant enzyme suppression, elevated ROS, membrane instability, and haemolysis. Additionally, HepG2 transcriptomics predicts INH-affected pathways, validated by erythrocyte assays showing enzymatic impairment and oxidative damage. This links liver transcriptional changes to blood cell dysfunction, bridging computational modelling with cross-system mechanisms. This integrated evidence links hepatic metabolic responses to systemic redox effects in blood cells, advancing understanding of INH-induced adverse reactions and paving the way for targeted TB therapy interventions to reduce side effects.

To further strengthen the study and provide additional mechanistic insights, future work could validate INH-induced OS and erythrocyte damage *in vivo*, assess gene expression/antioxidant enzymes in treated erythrocytes, and use inhibitors/silencing for hub genes (SOD1, GPX8, HMOX1). Although amlodipine reduced INH-induced MCV elevation (indirect Ca^2+^ evidence), direct intracellular Ca^2+^ measurements remain a key limitation. These experiments would build upon the resilient *in vitro* findings, enhancing understanding and translational potential within the scope of the study.

## 5 Conclusion

This study uncovers the molecular basis of INH-induced OS, highlighting disruptions in antioxidant enzymes and signalling pathways. Drug-triggered calcium influx causes RBC membrane damage and haemolysis, while calcium channel inhibition offers protective effects as a potential TB therapy adjunct.

## Data Availability

Publicly available datasets were analyzed in this study. This data can be found here: The dataset GSE168473 used in this study are available in the Gene Expression Omnibus (GEO) database (https://www.ncbi.nlm.nih.gov/geo/).
