# Supplementary material for: Isoniazid subverting erythrocyte homeostasis: implications for tuberculosis therapy
Source: Front Pharmacol. 2026 Feb 9;17:1721617. doi: 10.3389/fphar.2026.1721617 (PMC12926460; doi:10.3389/fphar.2026.1721617)
Supplement: Supplementary file 1 [file DataSheet1.pdf]

Supplementary Tables 1-2

Isoniazid subverting erythrocyte homeostasis: implications for tuberculosis therapy

Running title: INH toxicity in erythrocytes

Muhammad Sikandar, Maria Fatima, Kashif Jilani, Li Xing

Table 1. Correlation of INH-induced transcriptomic disruption with clinical haematological toxicity.

Molecular finding in HepG2 transcriptomics	Erythrocyte experimentation	Clinical outcome (INH-related hematology)
Disruption of JUN/FOS (AP-1) and antioxidant enzyme (CAT, SOD, GPx) transcriptional regulation.	Functional, dose-dependent suppression of SOD, GPx, and CAT activity in erythrocytes.	**Loss of redox homeostasis:** Failure to clear circulating INH-induced reactive oxygen species (ROS) and metabolites (1, 2)
**G6PD** was identified as a critical hub gene in the OS disease-gene network.	Impaired GPx function due to lack of NADPH regeneration, leading to severe oxidative stress.	**Hemolytic anemia:** Direct molecular mechanism for the known susceptibility of G6PD-deficient patients to INH-induced hemolysis (3, 4)
Upregulation of inflammatory pathways (IL-17, TNF signaling).	Increased erythrocyte membrane instability, observed as MCV expansion and membrane blebbing.	**Hemolysis and anemia:** Premature destruction of red blood cells, leading to a drop in hemoglobin and red blood cell count (4)

Table 1. This table summarizes the integrated findings from HepG2 transcriptomic profiling, erythrocyte functional assays, and clinical hematology outcomes, revealing a multifaceted pathway of INH-induced oxidative stress (OS). Key observations include disruption of JUN/FOS (AP-1) transcription factors and downregulation of antioxidant enzymes (CAT, SOD, GPx), leading to dose-dependent suppression of their activity, loss of redox homeostasis, and failure to clear INH-generated ROS and metabolites. G6PD emerges as a central hub gene, with impaired GPx function due to NADPH deficiency exacerbating OS, which directly links to hemolytic anemia susceptibility in G6PD-deficient patients via membrane instability (MCV expansion, blebbing), premature RBC destruction, hemoglobin decline, and upregulation of inflammatory pathways (IL-17, TNF signaling), consistent with prior clinical reports.

Table 2. Longitudinal hematological changes in pulmonary tuberculosis (PTB) patients during anti-tuberculosis therapy (ATT).

Patient cohort & design	Materials and methods	Key erythrocyte-related results	References
Longitudinal prospective study of newly diagnosed PTB patients.	Patients were followed for 6 months. Hematological parameters (RBC, Hgb, Hct, MCV, RDW) were measured at baseline, 2 months (intensive phase), and 6 months.	Significant increase in RDW (31.1% high at 2m vs 13.5% at baseline). Significant decrease in Hgb and Hct after 2 months of ATT. MCV showed a significant rise at 6 months (20.9% high).	(5)
Retrospective analysis of PTB patients stratified by anemia status.	Biochemical and hematological profiling at baseline and day 60 of ATT. Multidimensional statistical analysis (PCA and hierarchical clustering) was used to define inflammatory biosignatures.	88.9% prevalence of anemia at baseline. Anemia was linked to a distinct inflammatory profile (high CRP, ESR). Persistent inflammation and suppressed Hgb levels were observed even after 60 days of therapeutic dosing.	(6)
Longitudinal study of 168 newly diagnosed PTB patients.	Hematological parameters (RBC count, Hgb, Hct, MCV, RDW) were determined before and after the 2-month intensive phase of ATT (including INH).	Significant decreases in Hemoglobin (Hgb) and Hematocrit (Hct) were observed after 2 months of treatment. A significant increase in Red Cell Distribution Width (RDW) was noted, indicating increased variation in red blood cell size and a direct impact of therapeutic INH-containing dosing on erythrocyte indices. The RDW change	(7)

		suggests a shift in the population.	
Prospective study on hematological and haemostasis parameters.	Patients were evaluated at baseline and after 2 months of ATT. Correlation between inflammatory markers (ESR) and anemia recovery.	While anemia generally improved as inflammation (ESR) subsided, a subset of patients showed persistent or worsening erythrocyte indices, attributed to the side effects of the intensive phase regimen.	(8)

Table 2. This table compiles key findings from four clinical studies examining erythrocyte parameters in newly diagnosed PTB patients undergoing standard ATT regimens containing isoniazid (INH). Longitudinal assessments consistently reveal significant declines in hemoglobin (Hgb) and hematocrit (Hct) after the 2-month intensive phase, alongside marked increases in red cell distribution width (RDW), indicative of erythrocyte heterogeneity, membrane instability, and premature destruction. Elevated mean corpuscular volume (MCV) at later timepoints (6 months) further suggests compensatory reticulocytosis or direct INH-mediated toxicity.

References

1. Chen Y, Xue P, Hou Y, Zhang H, Zheng H, Zhou T, et al. Isoniazid suppresses antioxidant response element activities and impairs adipogenesis in mouse and human preadipocytes. Toxicology and applied pharmacology. 2013;273(3):435-41.
2. Lei S, Gu R, Ma X. Clinical perspectives of isoniazid-induced liver injury. Liver Research. 2021;5(2):45-52.
3. Mintzer DM, Billet SN, Chmielewski L. Drug‐induced hematologic syndromes. Advances in hematology. 2009;2009(1):495863.
4. Goldman AL, Braman SS. Isoniazid: a review with emphasis on adverse effects. Chest. 1972;62(1):71-7.
5. Reta B, Mohammed AE, Tesfaye Kiya G, Adissu W, Shenkute TY. Impact of anti-tuberculosis treatment on hematological parameters in newly diagnosed tuberculosis patients at Jimma town: a longitudinal prospective study. Ann Med Surg (Lond). 2023;85(8):3887-93.
6. Gil-Santana L, Cruz LA, Arriaga MB, Miranda PF, Fukutani KF, Silveira-Mattos PS, et al. Tuberculosis-associated anemia is linked to a distinct inflammatory profile that persists after initiation of antitubercular therapy. Scientific reports. 2019;9(1):1381.
7. Kassa E, Enawgaw B, Gelaw A, Gelaw B. Effect of anti-tuberculosis drugs on hematological profiles of tuberculosis patients attending at University of Gondar Hospital, Northwest Ethiopia. BMC hematology. 2016;16(1):1.
8. Kutiyal AS, Gupta N, Garg S, Hira HS. A study of haematological and haemostasis parameters and hypercoagulable state in tuberculosis patients in northern India and the outcome with anti-tubercular therapy. Journal of clinical and diagnostic research: JCDR. 2017;11(2):OC09.
